# The Regulatory Mechanism of Water Activities on Aflatoxins Biosynthesis and Conidia Development, and Transcription Factor AtfB Is Involved in This Regulation

**DOI:** 10.3390/toxins13060431

**Published:** 2021-06-21

**Authors:** Longxue Ma, Xu Li, Xiaoyun Ma, Qiang Yu, Xiaohua Yu, Yang Liu, Chengrong Nie, Yinglong Zhang, Fuguo Xing

**Affiliations:** 1 Institute of Food Science and Technology, Chinese Academy of Agricultural Sciences, Beijing 100193, China; longxuem@foxmail.com (L.M.); lixu@caas.cn (X.L.); xiaoyunma29@foxmail.com (X.M.); 2 Qingdao Tianxiang Foods Group Co., Qingdao 266737, China; yuqiang@tianxianggroup.cn (Q.Y.); yuxiaohua@tianxianggroup.cn (X.Y.); 3 School of Food Science and Engineering, Foshan University, Foshan 528231, China; liuyang@fosu.edu.cn (Y.L.); niecr@126.com (C.N.); 4 Shandong Institute of Commerce and Technology, Jinan 250103, China

**Keywords:** water activity, aflatoxin biosynthesis, conidia development, regulatory mechanism, AtfB

## Abstract

Peanuts are frequently infected by *Aspergillus* strains and then contaminated by aflatoxins (AF), which brings out economic losses and health risks. AF production is affected by diverse environmental factors, especially water activity (*a*_w_). In this study, *A. flavus* was inoculated into peanuts with different *a*_w_ (0.90, 0.95, and 0.99). Both AFB_1_ yield and conidia production showed the highest level in *a*_w_ 0.90 treatment. Transcriptional level analyses indicated that AF biosynthesis genes, especially the middle- and later-stage genes, were significantly up-regulated in *a*_w_ 0.90 than *a*_w_ 0.95 and 0.99. AtfB could be the pivotal regulator response to *a*_w_ variations, and could further regulate downstream genes, especially AF biosynthesis genes. The expressions of conidia genes and relevant regulators were also more up-regulated at *a*_w_ 0.90 than *a*_w_ 0.95 and 0.99, suggesting that the relative lower *a_w_* could increase *A. flavus* conidia development. Furthermore, transcription factors involved in sexual development and nitrogen metabolism were also modulated by different *a*_w_. This research partly clarified the regulatory mechanism of *a*_w_ on AF biosynthesis and *A. flavus* development and it would supply some advice for AF prevention in food storage.

## 1. Introduction

Peanut is an important economical crop for oil production and nutritious addition in human consumption. However, aflatoxigenic *Aspergillus* strains infection and aflatoxins (AF) contamination bring out immense human health risks and huge economic losses for the peanut industry. AF are the polyketide-derived furanocoumarins with strong carcinogenicity that associated with both acute and chronic toxicity for animals and humans [1]. More than 28% hepatocellular carcinoma cases are induced by AF contamination in the world [2]. Among the diverse AF, aflatoxin B_1_ (AFB_1_), as the most toxic and dangerous one, is usually high-level-produced by some aflatoxigenic *Aspergillus* strains [3]. Therefore, investigating *A. flavus* growth and metabolism, especially AF biosynthesis, is extremely essential for controlling AF contamination.

The AF biosynthesis and fungal development of *A. flavus* are affected by diverse environmental factors, such as water activity (*a*_w_), temperature, pH, carbon source, nitrogen source, and oxidative stress. Based on the definition of U. S. Food and Drug Administration (FDA), *a*_w_ of a food is the ratio between the vapor pressure of the food itself, when in a completely undisturbed balance with the surrounding air media, and the vapor pressure of distilled water under identical conditions. So, *a*_w_ as a parameter to measure the freely available water in food or substrate is directly related to the food microbial growth in a specific condition [4]. More importantly, *a*_w_ was regarded as a central environmental factor, and could co-modulate the fungal development and toxin production of *Aspergillus* spp. with other environmental factors [5,6,7]. Previous studies reported that the proper *a*_w_ conditions for AF biosynthesis were dependent on the other environmental factors, for example, temperature, pH, light, and especially culture substrates [5,8,9]. However, few researchers focused on the effect of peanut substrates with different *a*_w_ on *A. flavus* development and AF production.

As the most important characters of *A. flavus*, AF biosynthesis has been well researched in past decades. More than 20 structural genes, located in the 80-kb AF cluster, are involved in the series enzymatic reactions, and transform acetyl-CoA to AFB_1_, AFB_2_, AFG_1_, and AFG_2_ [10]. Two pathway specific regulators, DNA binding protein AflR and transcriptional co-activator AflS, are affected by other regulators or environmental factors, and then modulate the structural genes’ transcriptions [9,11]. AF production are also regulated by plenty of global regulators including the velvet complex, MAPK pathway factors, oxidative-stress-related regulators, G-protein receptors, oxylipin proteins, as well as many oxidative stress transcription factors (TFs) [10,12]. All AF biosynthetic enzymes and AF regulators constitute an extremely complicated system, and diverse environmental factors affect AF production by adjusting the expression of the AF regulatory system. In previous studies, the expression of AF structural genes could have been affected by diverse *a*_w_, and the ratio of *aflS*/*aflR* was more down-regulated in *a*_w_ 0.99 than *a*_w_ 0.96 [6,8,9,13]. However, the mechanism of *a*_w_ on AF biosynthesis regulation is still unclear.

Transcriptome analysis is regarded as an effective and efficient method to discover the new regulatory mechanisms. In previous studies, the optimal *a*_w_ for AF biosynthesis were in the range of 0.90–0.99 at the different environmental combinations [6,8,9,13]. In this study, the *a*_w_ of shelled peanuts were adjusted as 0.90, 0.95, 0.99, and the AF production and fungal growth were confirmed at different *a*_w_. By comprehensive transcriptional analysis, AF cluster genes, conidia development genes, and several TFs were significantly up-regulated at *a*_w_ 0.90, and AtfB was regarded as the critical TFs for AF regulation in diverse *a*_w_. This work contributes to better understanding of the regulatory mechanism of *a*_w_ on *A. flavus* development and AF biosynthesis, and it is helpful to reduce the AF contamination in peanuts storage.

## 2. Results

### 2.1. Water Activity Affects the Conidia Production and the AFB_1_ Production of A. flavus in Peanuts

**Figure 1:**
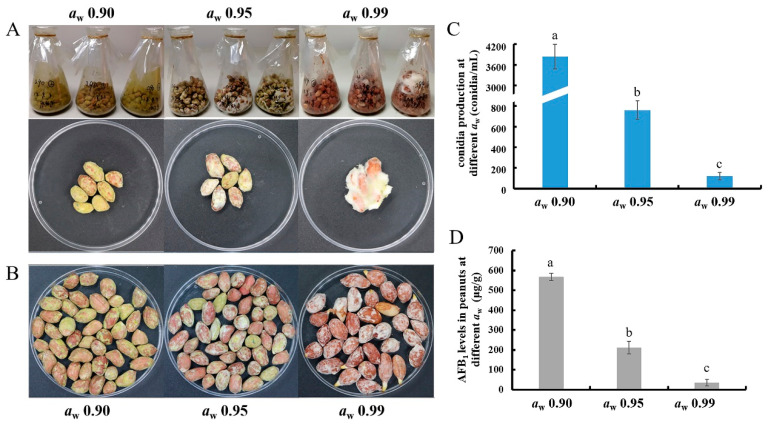
The differences of AFB_1_ yield and conidia production in peanuts in different *a*_w_. (**A**) The inoculated peanuts with different *a*_w_ were placed in flasks for 10 days’ cultivation, and (**B**) 25 g treated peanuts were used for AFB_1_ detection and conidia examination. (**C**) *A. flavus* conidia from peanuts were counted by hemocytometer, and (**D**) AFB_1_ levels in different *a*_w_ peanuts were detected by HPLC. All experiments were performed in three independent biological replicates, and results were represented as means ± SD. Samples marked with different letters show a significant difference at *p* < 0.05.

After 10 days cultivation, almost all of the peanuts at *a*_w_ 0.90 were covered by the green conidia and mycelia, while the conidia and the green color were significantly less at *a*_w_ 0.95 (Figure 1A,B). At *a*_w_ 0.99, peanuts were only coated by white mycelia, but without obvious conidia production (Figure 1A,B). After counting the peanut-washed suspensions by hemocytometer, the conidia concentrations were more than 3800 conidia/mL in *a*_w_ 0.90, and less than 800 conidia/mL in *a*_w_ 0.95, but few conidia were in *a*_w_ 0.99 treatment (Figure 1C). The AFB_1_ levels in contaminated peanuts in different *a*_w_ treatments were also examined (Figure 1D). At *a*_w_ 0.90, 568 μg/g AFB_1_ were detected, while AFB_1_ levels were significantly decreased at *a*_w_ 0.95 and 0.99, with 212 μg/g and 36 μg/g, respectively (Figure 1D). So, these results concluded that in shelled peanuts with *a*_w_ 0.90–0.99, the conidia development and AFB_1_ production of *A. flavus* were increased in the relatively lower *a*_w_ conditions.

### 2.2. Transcriptome Analyses of the A. flavus Genes Expressions in Different Water Activity

**Figure 2:**
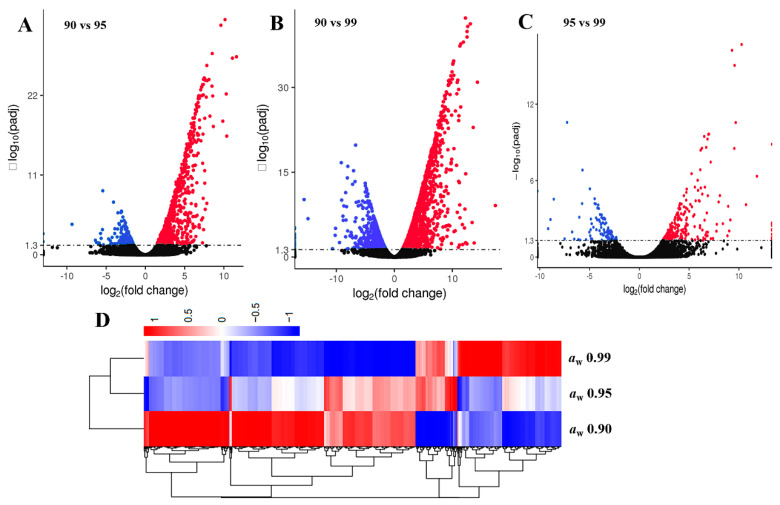
Transcriptomic analyses of *Aspergillus flavus* in different *a*_w_. The volcano plots of the pairwise comparisons in (**A**) *a*_w_ 0.90 vs. 0.95, (**B**) *a*_w_ 0.90 vs. 0.99, and (**C**) *a*_w_ 0.95 vs. 0.99. Up-regulated and down-regulated genes were showed with red spots and blue spots, respectively, and no significantly changed genes were presented with black spots. (**D**) Cluster analysis of DEGs in diverse *a*_w_. Up-regulated and down-regulated genes were represented in red and blue, respectively. The transcriptomic analyses were performed in three independent biological replicates.

**Figure 3:**
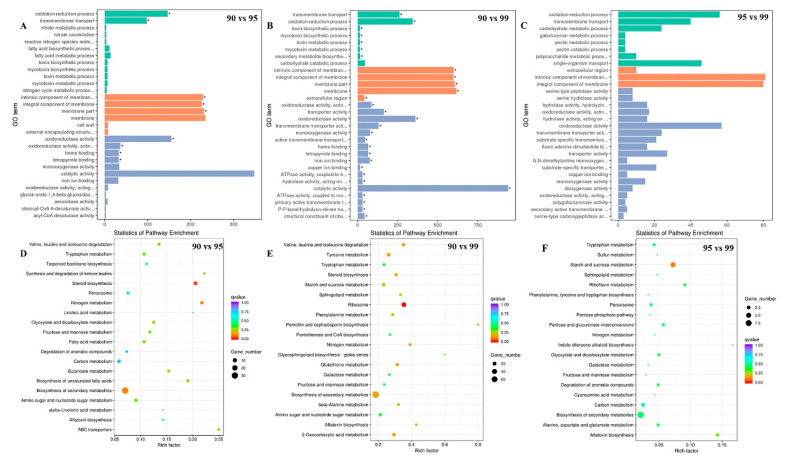
GO annotation and KEGG enrichment of DEGs in different *a*_w_. Bar charts demonstrated the GO-enriched results in comparisons of (**A**) *a*_w_ 0.90 vs. 0.95, (**B**) *a*_w_ 0.90 vs. 0.99, and (**C**) *a*_w_ 0.95 vs. 0.99. The number of enriched genes and the names of GO terms are showed in *X*-axis and *Y*-axis, respectively. Biological process, cellular components, and molecular function were represented by the green bars, orange bars, and blue bars, respectively. The top 20 enriched KEGG pathways were showed in (**D**) *a*_w_ 0.90 vs. 0.95, (**E**) *a*_w_ 0.90 vs. 0.99, and (**F**) *a*_w_ 0.95 vs. 0.99. The rich factors and the pathway names are showed in *X*-axis and *Y*-axis, respectively. The size of spots represented the number of enriched genes, and different colors described the *q*-value.

To explore the regulatory mechanisms of *a*_w_ on *A. flavus* development and AF biosynthesis in peanuts, transcriptome analyses were performed. A total of 14,472 genes were mapped to the *A. flavus* NRRL3357 genome and 671 novel genes were identified from the transcriptome data. Compared with *a*_w_ 0.95 treatment, 834 DEGs of *A. flavus* in *a*_w_ 0.90 were up-regulated, while 148 DEGs were down-regulated (Figure 2A). A total of 2667 DEGs with 1760 up-regulated and 907 down-regulated were identified in a comparison of *a*_w_ 0.90 vs. 0.99 (Figure 2B). In a comparison of *a*_w_ 0.95 vs. 0.99, 233 genes were increased, and 95 genes were decreased (Figure 2C). A heat map of the DEGs clustering also showed the obviously differential expression pattern among the three *a*_w_ conditions, of which the most genes were up-regulated in *a*_w_ 0.90 treatment, while two thirds of the genes were down-regulated at *a*_w_ 0.99 (Figure 2D). GO annotation analysis of the comparisons of *a*_w_ 0.90 vs. 0.95 and *a*_w_ 0.90 vs. 0.99 found that DEGs were enriched in oxidation-reduction process and transmembrane transport in biological process, the intrinsic component of the membrane, the integral component of the membrane, the membrane part, the membrane in the cellular component, and catalytic activity in molecular function (Figure 3A,B). DEGs in *a*_w_ 0.95 vs. 0.99 were enriched in similar GO items, such as oxidation-reduction process, single-organism transport, transmembrane transport in biological process, the intrinsic component of membrane, the integral component of the membrane in the cellular component, and oxidoreductase activity in molecular function (Figure 3C). KEGG pathway annotation revealed DEGs of the different *a*_w_ comparisons were mainly enriched in biosynthesis of secondary metabolites, steroid biosynthesis, nitrogen metabolism, ribosome, valine, leucine and isoleucine degradation, and starch and sucrose metabolism (Figure 3D–F).

### 2.3. Expression Changes of AF Cluster Genes in Different a_w_ Conditions

Based on transcriptomic analyses, the transcriptional variations of AF cluster genes were listed in Table 1. In comparison of *a*_w_ 0.90 vs. 0.95, 24 of 34 AF biosynthetic genes were significantly up-regulated. The 25 genes of the AF cluster were apparently increased in *a*_w_ 0.90 than *a*_w_ 0.99, and 15 AF biosynthesis genes were significantly up-regulated in *a*_w_ 0.95 than *a*_w_ 0.99. Among these genes, *aflV*, *aflO*, *aflI*, *aflLa*, and *aflL* showed the most obviously increased in *a*_w_ 0.90, but the expression of initial steps genes, *aflA* and *aflB*, were not increased in comparisons of *a*_w_ 0.90 vs. 0.95 and *a*_w_ 0.95 vs. 0.99. The expressions in different *a*_w_ treatments of the pathway-specific regulators, AflR and AflS, showed up-regulations, but were not significantly changed in *a*_w_ 0.90 vs. 0.95 and *a*_w_ 0.95 vs. 0.99. All these results suggested that transcriptional expressions of the AF cluster genes could be affected by different *a*_w_ levels.

### 2.4. Varying Expressions of Diverse Regulator-Associated AF Biosynthesis in Different a_w_ Conditions

The expression changes of AF biosynthesis-related regulators were listed in Table S1. The majority regulators’ expressions, such as the velvet complex genes, the MAPK pathway genes, and the GPCRs genes, were not significantly different in diverse *a*_w_ conditions. However, the bZIP TF, AtfB, was obviously changed at different *a*_w_ conditions, and the *atfB* levels showed to be significantly up-regulated in comparisons of *a*_w_ 0.90 vs. 0.99 and *a*_w_ 0.95 vs. 0.99 (Table S1). The other AF production-related TFs were not noticed any differently at different *a*_w_ (Table S1). The transcriptional expressions of the oxylipin genes *ppoB* were significantly up-regulated at lower *a*_w_, while *ppoA* and *ppo*C showed similar levels in different *a*_w_ comparisons (Table S1). The calcium-binding protein caleosin gene, *AfPXG*, and the cAMP-dependent protein kinase gene, *pkaC*, were not apparently changed in *a*_w_ 0.90 vs. 0.95, whereas they showed significantly increased levels in *a*_w_ 0.90 vs. 0.99 and *a*_w_ 0.95 vs. 0.99 (Table S1). Concerning SakA, homologous with HogA in *Saccharomyces cerevisiae*, its transcriptional expressions were down-regulated at the lower *a*_w_, but significantly changed only in comparison of *a*_w_ 0.90 vs. 0.99 (Table S1).

### 2.5. Different Expression of the Genes Controlling Conidia Production in Different Water Activities

The transcriptional expressions of several conidia developmental and regulatory genes were also analyzed in transcriptome analyses (Table 2). Six conidial development proteins, including conidiation-specific family protein (AFLA_044790), conidiation proteins Con6 and Con10, conidial hydrophobin RodA and RodB, and conidial pigment biosynthesis oxidase Arb2, showed significantly up-regulated transcription in the lower *a*_w_ conditions (Table 2). However, conidial-pigment-biosynthesis-related gene *arp1* and conidiophore-development-related gene *hymA* showed no difference at different *a*_w_ (Table 2). Several pieces of research reported that the velvet complex and the developmental signal biosynthesis protein FluG could affect the conidia production. However, *veA*, *laeA*, *velB*, and *fluG* showed similar expression in diverse *a*_w_ (Table 2). The transcriptional expressions of the developmental regulator FlbA and the conidiation-related TFs, FlbC and StuA, were also not significantly different at *a*_w_ 0.90, 0.95, and 0.99 conditions (Table 2). However, the C_2_H_2_ type conidia developmental TF gene *brlA* and the developmental regulator genes, *vosA* and *wetA*, showed to be significantly more up-regulated at *a*_w_ 0.90 than in *a*_w_ 0.95 and 0.99 (Table 2). Taken together, the expressions of conidia developmental proteins and their regulators could be affected by different *a*_w_ conditions.

### 2.6. The Effects of Diverse Water Activities on Transcription Factors

The TFs’ expressions in different *a*_w_ were additionally analyzed in this study. In a total of 271 TFs (annotated in this transcriptome data), 29 transcriptional factors showed significant variations in the comparison of *a*_w_ 0.90 vs. 0.99 (Table 3). Among them, 20 genes were significantly up-regulated at *a*_w_ 0.90, while the other nine genes were significantly down-regulated. With the exception of the two mentioned TFs, BrlA and AtfB, the TFs, including LeuB, RosA, NosA, AbaA, and MeaB, were also significantly increased at *a*_w_ 0.90 compared to *a*_w_ 0.99. In the comparison of *a*_w_ 0.90 vs. 0.95, the expressions of TF genes, AFLA_029620 (*abaA*), AFLA_040300, AFLA_082850 (*brlA*), and Novel 00457 were up-regulated at *a*_w_ 0.90. In the comparison of *a*_w_ 0.95 vs. 0.99, only *nosA*, *atfB*, and *brlA* levels were increased. So, several TFs genes were affected by *a*_w_ conditions, and further regulated the transcriptions of downstream genes.

### 2.7. RT-qRCR Analyses of Genes Expressions Involved in AF Biosynthesis and Conidia Development

**Figure 4:**
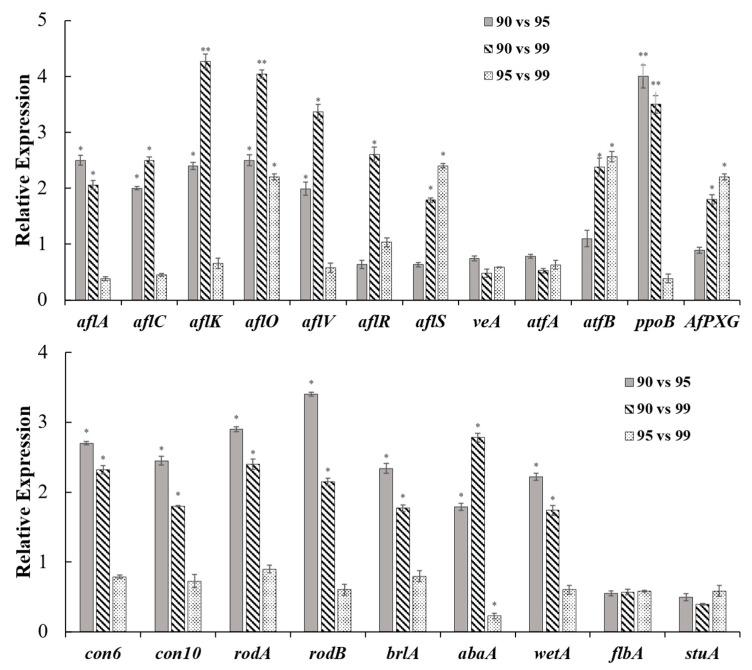
Transcriptional expression analyses of diverse genes by RT-qPCR. The RT-qPCR analysis of (**A**) AF biosynthesis-related genes and (**B**) conidia developmental genes in different *a*_w_ conditions. The different *a*_w_ comparisons were showed as diverse bars. Three independent biological replicates were performed in each condition, and data were presented as means ± SD. *t* tests were applied for significance analyses with * *p* < 0.05 and ** *p* < 0.01.

RT-qPCR was performed for confirming the transcriptome results. Similar with transcriptome data, *aflA* and *aflC* were up-regulated at *a*_w_ 0.90 compared with *a*_w_ 0.95 and 0.99, and *aflK*, *aflO*, and *aflV* were more drastically increased. Additionally, *aflO* in comparison to *a*_w_, 0.90 vs. 0.99 showed the biggest difference with 4.04-log_2_FoldChange. The *aflR* was only significantly changed in *a*_w_ 0.90 vs. 0.99, while *aflS* levels were increased at *a*_w_ 0.90 and 0.95 compared to *a*_w_ 0.99 (Figure 4A). The transcripts of *atfB*, *ppoB*, and *AfPXG* were significantly up-regulated under the lower *a*_w_ conditions, but the expressions of *veA* and *atfA* were not significantly changed (Figure 4A). The conidia developmental genes, *con6*, *con10*, *rodA*, and *rodB*, were significantly up-regulated at *a*_w_ 0.90 compared with *a*_w_ 0.95 and 0.99. The conidial regulators, *brlA*, *abaA*, and *wetA* were also obviously increased at *a*_w_ 0.90, but the other two regulators, *flbA* and *stuA*, had no obvious variations (Figure 4B). In order to verify our results, we also investigated these genes’ expressions in other *Aspergillus* strains at different *a_w_* conditions. In *A. flavus* CA14, all AF cluster genes’ expressions were similar with *A. flavus* NRRL3357, but with the exception of *atfB*, the expression of *atfA* was also up-regulated in *a_w_* 0.90 compared than *a_w_* 0.99 (Figure S1). In *A. flavus* ACCC32656, both atfA and atfB were increased in the lower *a_w_* conditions, but the *aflA* and *aflC* were not significantly changed (Figure S1). For the conidiation, the conidial genes’ expressions were similar in different strains, while the *wetA* in ACC32656 were not significantly varied in diverse *a_w_* conditions.

## 3. Discussion

In this paper, the *a*_w_ 0.90 of peanuts showed the maximum AFB_1_ production after 10 days cultivation (Figure 1D). Abdel-Hadi et al. found that *A. flavus* in peanuts would produce the maximum amounts of AFB_1_ at *a*_w_ 0.90–0.95 after 3 weeks storage [13]. Liu et al. indicated that AFB_1_ levels were obviously increased in *a*_w_ 0.95, followed by *a*_w_ 0.90, but were suppressed in *a*_w_ 0.99 [6]. The relatively low peanut *a*_w_ could be suitable for AF production, and *a*_w_ 0.99 could not be a proper condition for AF biosynthesis. We believed that the condition of *a*_w_ 0.99 could be a stress signal for *A. flavus*. However, in other studies, the results could be opposite. Zhang et al. found that *A. flavus* produced more AFB_1_ in *a*_w_ 0.99 than at *a*_w_ 0.93 in YES medium, and Medina et al. noticed that AFB_1_ levels of maize were lower in *a*_w_ 0.91 than 0.99 [8,9]. It seems like the suitable *a*_w_ levels could be varied depending on diverse substrates. Different temperatures also influence the optimum *a*_w_ for AF biosynthesis. The optimal *a*_w_ for AF biosynthesis was 0.92 upon 28 °C, while it increased to 0.96 at the lower temperature [14]. Further, the effect of *a*_w_ on AF production was apparently modulated by the stages of cultivation, maturity, and storage [15]. Strain-specificity is another important reason for different AF productions, such as *A. flavus* CA14 showing the highest AF production in *a_w_* 0.95 [6], but *A. flavus* NRRL3357 showing the most AF levels in *a_w_* 0.90. Taken all this, it is concluded that *a_w_* is a crucial factor for AF biosynthesis, and the effect of *a*_w_ on AF production is dependent on other environmental factors, such as temperature, substrates, pH, cultivation time, and different strains. Because of the diverse experiment conditions, it is hard to get a consistent result. So, in this study, we focused our research on the regulatory mechanism of *a_w_* on AF biosynthesis.

AF cluster gene expressions are directly related to AF biosynthesis. There are some studies reporting the variations of AF gene expression in different *a_w_*. Most AF genes had higher expression levels at lower *a_w_* [6], and *aflD* showed higher expression at aw 0.90 [13]. In this study, we examined the transcriptional expressions of AF cluster genes by RNA-seq and RT-qPCR analyses (Table 1 and Figure 4A). The majority of genes (27/34) in AF clusters were significantly up-regulated at the relatively lower *a*_w_ (90 and 95) (Table 1). These results differed from previous reports [16,17], but were similar with Liu et al. [6]. The AF biosynthetic initial-genes, *aflA*, *aflB*, *aflC*, and *aflD*, showed slight or moderate variations at different *a*_w_ (Table 1 and Figure 4A). Abdel-Hadi et al. suggested the initial step gene *aflD* was a good indicator of AFB_1_ production [13]. However, in our study, *aflD* expressions in *a*_w_ 0.95 vs. 0.99 were not significantly different, and were mildly changed in *a*_w_ 0.95 vs. 0.99 and *a*_w_ 0.95 vs. 0.99 (Table 1). Ehrlich suggested that the later stages of AFB_1_ biosynthesis were more critical than the beginning stages [18]. In our study, the AF cluster genes in medium or later stages, such as *aflI*, *aflO*, *aflP*, *aflQ*, *aflK*, and *aflV*, showed more drastic variations in different *a*_w_ conditions. All the above information indicated that AF biosynthesis was influenced by different *a*_w_, especially the biosynthetic process from norsolorinic acid (NOR) to O-methylsterigmatocystin (OMST).

Transcriptions of AF biosynthetic genes are mainly regulated by the cluster-specific regulators, AflR and AflS, which directly bind to the promoter region of AF cluster genes [19]. In our research, *aflR* and *aflS* levels in *A. flavus* NRRL3357 and ACCC32656 showed the moderate increases at *a*_w_ 0.90 vs. 0.95, while no significant variations of *aflR* and *aflS* were noticed in the other two *a*_w_ comparisons (Table 1 and Figure 4A). However, in *A. flavus* CA14, *aflR* and *aflS* were increased in *a_w_* 0.90 compared with *a_w_* 0.99 (Figure S1), suggesting the AF cluster-specific regulators might be affected in different strains upon the diverse *a_w_*. There are also many studies that found that the ratio of *aflS*/*aflR* should have the closer correlation with AF productions [9,11,17]. However, in this research, the ratios of *aflS*/*aflR* were still similar in different *a*_w_ treatments. So, the transcriptional changes of AF structural genes could not be only caused by the changes of *aflR* and *aflS*, but other regulators could play more important roles.

Furthermore, there are some papers reporting that the expressions of AF cluster genes were influenced by different environmental factors. However, few of them focused on how *a_w_* affected AF genes’ expression, and what the critical regulator response to *a_w_* is. In this study, to deeply investigate the reasons of AF gene variations in different *a_w_*, the comprehensive transcriptomic analysis was performed, and the oxidation-stress-related TFs, AtfA, AtfB, AP-1, MsnA, MtfA, and SrrA, were also examined, which could control the AF cluster gene transcriptions by directly binding [12,20,21]. However, in this study, the above TF genes, with the exception of AtfB, showed similar transcriptional expressions at different *a*_w_ (Table S2 and Figure 4A). The *atfB* expression was significantly different in different *a*_w_ conditions (Table S2 and Figure 4A), suggesting AtfB should be a key responder of *a*_w_ conditions. AtfB, as a member of CREB family protein, could recognize the CRE binding sites (5′-TG/TACGTC/AA-3′), and start the target gene transcript [12]. In *A. parasiticus*, in the upstream noncoding regions of *aflB*, *aflD*, *aflM*, *aflO*, and *aflR*, were found the CRE sites, which could be directly bound by AtfB [22]. So, their transcriptional expressions were positively correlated with *atfB* expression. Suppression of AtfB could significantly reduce the AF genes’ mRNA levels and the AF production [23]. Similarly, in this study, significantly more down-regulation of *atfB* was found at *a*_w_ 0.95 and 0.99 than *a*_w_ 0.90; subsequently, most AF genes and AF productions also were decreased at the higher *a*_w_ conditions. In recent research, AtfB was suppressed by methyl jasmonate, and subsequently, down-regulated AF gene expressions [24]. So, AtfB is a critical regulator for sensing and response to environmental changes, and then could modulate downstream genes, such as AF cluster genes in *A. flavus*. Additionally, we also tested the *atfB* expression in other *Aspergillus* strains, of which the *atfB* in *A. flavus* CA14 and *A. flavus* ACCC 32656 were significantly up-regulated in *a_w_* 0.90 (Figure S1). All these results that confirmed the differential expression of *atfB* in different *a*_w_ treatments might play a vital role in the changes of AF genes’ expressions and AF production.

The environmental signals could be sensed by the membrane protein, transferred by the phosphorylation signal, and responded to by TFs. For example, the oxidation stresses up-regulate SAPK/MAPK signaling cascade, and then activate AtfB for binding to the target promoters [12]. In this study, *sakA2* (AFLA_099500), a kinase of MAPK pathway, is slightly down-regulated in *a*_w_ 0.90 vs. 0.99, suggesting it could be affected by different *a_w_* conditions (Table S2). However, we did not find other differential transcriptional expressions of MAPK genes in different *a*_w_ conditions (Table S2). It could be explained that the MAPK cascade transmits the signal by phosphorylation, and the effect of different *a*_w_ on MAPK genes could be at a post-transcriptional level. *pkaC*, an encoding cAMP-dependent protein kinase catalytic subunit, was significantly more down-regulated at *a*_w_ 0.99 than at *a*_w_ 0.90 and 0.95 (Table S2). The cAMP/PKA pathway can also regulate AF biosynthesis partly through AtfB [23,25], and AtfB responds to carbon sources and oxidative stress through the cAMP pathway [22]. It is a reasonable hypothesis that *pkaC* levels are modulated at different *a*_w_ levels, and then affect AtfB expression by the cAMP signaling pathway.

In previous studies, the conidia production and conidia germination of *Aspergillus* strains and *Penicillium* strains were significantly affected by different *a*_w_ levels [26,27]. We also noticed that the apparently decreased conidia production at *a*_w_ 0.99 in peanuts (Figure 1C), and transcriptions of conidial genes, were also significantly decreased at *a*_w_ 0.99 (Table 2 and Figure 4B). The *con6* and *con10*, as the representatives of conidiation genes, are conserved in filamentous fungi and preferentially expressed during the conidia development [28]. In *A. nidulans*, *conF* (homologous with *con6*) and *conJ* (homologous with *con10*) were increased with light exposure [29]. Similarly, their expressions at different *a*_w_ were obviously changed (Table 2 and Figure 4B), suggesting that *con* genes may be affected by diverse environmental factors. RodA and RodB, as the hydrophobin proteins, help conidia dispersion and attachment [30], and their transcriptions were also increased at the lower *a*_w_ (Table 2 and Figure 4B). It is also noticed that the conidial pigment-related gene, *arb2*, was significantly down-regulated in *a*_w_ 0.99 (Table 2). It could partly explain why the green color was faded in the higher *a*_w_ conditions (Figure 1A,B).

Conidia-relevant regulators, BrlA, AbaA, VosA, and WetA, were also significantly increased in *a*_w_ 0.90, and decreased in *a*_w_ 0.99 (Table 2 and Figure 4B). BrlA, as the C_2_H_2_ zinc finger TF, governs the *wetA* and *abaA* expressions, and positively regulates conidia production [31]. The transcript of *abaA* is promoted by BrlA in the middle stages of conidia development, and involved in the differentiation and functionality of phialides [32]. Lack of AbaA leads to the decreased and aberrant conidia production [33]. *wetA* is regulated by AbaA during the late phase of conidia development, and plays a role in the conidial wall component biosynthesis [34]. Based on previous research, deletion of any of the three genes could interfere with the conidial genes’ expression and conidial development. In this study, few conidia were produced at *a*_w_ 0.99, and conidiation-related genes were also significantly down-regulated. It is supposed that *a*_w_ might regulate conidia development through the BrlA-AbaA-WetA cascade. In addition, the *brlA* expressions of both *A. flavus* CA14 and *A. flavus* ACCC 32656 were significantly up-regulated in lower *a_w_*, but *wetA* in *A. flavus* ACCC 32656 showed no change in different treatments (Figure S1), suggesting that other regulators might be affected by *wetA* expression in *A. flavus* ACCC 32656. VosA is also a multifunctional regulator, interacting with VelB and VelC, and controls conidial trehalose amount and conidial germination in *A. fumigatus* [35,36]. We also noticed significantly increased *vosA* expression at *a*_w_ 0.90, but no obvious difference in other velvet complex genes (*veA*, *velB*, and *velC*). The other conidial regulators, FluG, FlbA, FlbC, FlbD, and StuA, [37], were not significantly regulated at diverse *a*_w_ (Table 2 and Figure 4B). Furthermore, AtfB was positively relevant with conidia production in *A. oryzae* [38], suggesting AtfB could also be a conidial regulator. In this study, AF production, conidia development, as well as *atfB* expression, showed similar changes in diverse *a*_w_ conditions, suggesting that AtfB might be a critical linker of fungal development and secondary metabolism.

**Figure 5:**
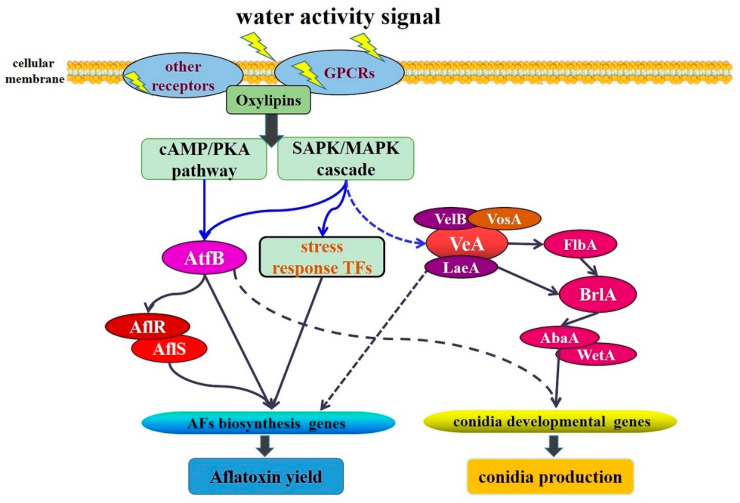
Hypothetical regulatory mechanism of *a*_w_ on AF biosynthesis and conidia development. The confirmed regulatory pathway and deduced regulatory pathway were presented as solid lines and dashed lines, respectively. TFs stands for transcription factors.

Taken together, the deduced regulatory pathway of different *a*_w_ effects on AF biosynthesis and conidia development were presented in Figure 5. As Figure 5 shows, different *a*_w_ signals affect cellular signaling pathways by modulating the expressions of GPCRs and oxylipins genes; then, several TFs, especially AtfB, are activated by SAPK/MAPK and cAMP/PKA pathways through the multistep phosphorelay systems [12,25]; the up-regulated AtfB can directly bind to the promoter regions of AflR, AflS, and AF biosynthetic genes, and subsequently enhance AF production [12,22]. BrlA, as the central regulator of conidiation, could be up-regulated by *a*_w_ 0.90, then motivate AbaA and WetA, and subsequently regulate conidial gene expressions. There are still a lot ambiguous specific regulations in this pathway, and more research is needed to clarify the regulatory mechanism of *a*_w_ on AF production and *A. flavus* development.

For better revealing of the transcriptional regulations in different *a*_w_, we also detected the expressions of diverse TFs. Among 271 annotated TFs, 29 TFs were significantly changed, including *leuB*, *rosA*, *nosA*, *abaA*, *meaB*, *brlA*, *atfB*, etc. (Table 3). NosA and RosA, as the Zn(II)_6_Cys_6_ class activators, are homologous with Pro1 in *Sordaria macrospora*, and regulate sexual development in *Aspergillus* [39]. However, RosA represses sexual development in the early stage, while NosA is necessary for primordium maturation [40]. The significant increase of *nosA* and *rosA* was observed at *a*_w_ 0.90 vs. 0.99, suggesting that sexual development of *A. flavus* may be affected by diverse *a*_w_ levels. MeaB as the methylammonium-resistant protein, is involved in nitrogen metabolite repression, and positively regulates sterigmatocystin production in *A. nidulans* [41]. However, in *A. flavus*, *meaB* was up-regulated at the higher *a*_w_ condition, and was negatively relevant with AF production (Table 3). LeuB/Leu3 participates in branched-chain amino acids biosynthesis, *gdhA* expression, as well as nitrogen metabolism, and physically interacts with AreA [42,43]. Moreover, by KEGG analysis, DEGs were obviously enriched in nitrogen metabolite (Figure 3). All information indicated that nitrogen metabolite of *A. flavus* in peanuts was also affected by diverse *a*_w_ levels.

## 4. Conclusions

In this study, *A. flavus* strain NRRL3357 was inoculated in peanuts with diverse *a*_w_ (0.90, 0.95, and 0.99). The changes of AFB_1_ yield and conidia production showed the highest level in *a*_w_ 0.90, followed by *a*_w_ 0.95, and the minimal level in *a*_w_ 0.99. Based on transcriptome data and RT-qPCR analyses, we noticed that (1) most of the AF biosynthesis genes were more up-regulated in *a*_w_ 0.90 than *a*_w_ 0.95 and 0.99; (2) the initial-step AF genes were slightly or moderately changed, while the middle- or later-step genes showed drastic responses to different *a*_w_ conditions; (3) several kinases, membrane proteins, and TFs were affected by different *a*_w_, and AtfB could be the central TF for regulating the transcriptional expressions of downstream genes, especially AF structural genes; (4) conidia development genes and the conidial regulator genes were up-regulated in *a*_w_ 0.90; (5) sexual-development-relevant TFs, NosA and RosA, and nitrogen-metabolite-relevant TFs, MeaB and LeuB, were significantly changed at diverse *a*_w_.

## 5. Materials and Methods

### 5.1. Fungal Strain and Conidia Suspension Preparation

*A. flavus* NRRL3357 and ACCC32656 were kindly provided by Professor Wenbing Yin (Institute of Microbiology, Chinese Academy of Sciences, Beijing, China). *A. flavus* CA14 was kindly provided by Professor Shihua Wang (Fujian Agriculture and Forestry University, Fujian, China). The strains were stored at −80 °C and re-cultivated on PDA medium (200 g potato, 20 g glucose, and 20 g agar in 1 L distilled water) at 28 °C in the dark. Conidia were harvested from PDA plates after 7 days inoculation by 0.01% Tween 20, and the suspension concentration was counted by hemocytometer, and was adjusted as 10^7^ conidia/mL.

### 5.2. Adjustment of Peanut Water Activities and Inoculation of A. flavus Conidia Suspension

The method of *a*_w_ adjusting was followed as that by Liu et al. with some modifications. The *a_w_* levels were detected by the Aqualab 4TE (Decagon Devices, Pullman, WA, USA), and the *a*_w_ curve of peanuts was performed in pre-experiment for accurately defining the amount of water added into the peanuts [6]. For adjusting the specific *a*_w_, 100 g of peanuts were put into zip-lock bags, irradiated with UV light for 2 h, and then the determined amount of water was added to them to obtain targeted a_w_ levels (a_w_ 0.90, 0.95, and 0.99). All treatments were placed in 4 °C overnight for the stable a_w_ levels.

Then these treated peanuts were transferred into the 500 mL sterile flasks, and incubated in 10 mL of the 10^7^ conidia/mL conidia suspension. Fungi in different *a*_w_ levels were cultivated at 28 °C for 10 days in the polyethylene boxes, which contained the glycerol-water solution for maintaining the relatively constant humidity. Peanut kernels without inoculating conidia suspension were prepared as a negative control. Each flask was shaken once a day. Three biological replicates were performed for all treatments.

### 5.3. Conidia Assessment and AFB1 Detection

After 10 days cultivation, 25 g of inoculated peanuts with different *a*_w_ were added 100 mL sterilized H_2_O, fiercely shaken for 30 min, filtered with non-woven fabric, and conidia of the solution was counted by a hemocytometer.

AFB_1_ concentration was detected by HPLC analysis. An amount of 25 g of peanut samples were finely grounded, 125 mL 70% methanol water and 5 g NaCl were added, and fiercely vibrated for 30 min. AFB_1_ extractions was purified by ToxinFast immunoaffinity columns as per the manufacturer’s instructions (Huaan Magnech Biotech, Beijing, China), and were examined by an Agilent 1220 Infinity Ⅱ HPLC system coupled with a fluorescence detector and a post-column derivation system (Huaan Magnech Biotech, Beijing, China). The excitation wavelength was 360 nm, and the emission wavelength was 430 nm. The HPLC system was matched with the Agilent TC-C18 column (250 mm × 4.6 mm, 5 μm particle size, Agilent). An amount of 20 μL AFB_1_ samples were injected each time, 70% methanol solution was the mobile phase, and the retention time was about 5.7 min. AFB_1_ standards were purchased from Sigma-Aldrich (St. Louis, MO, USA).

### 5.4. Total RNA Extraction

RNA samples for transcriptome analysis and RT-qPCR were performed three times by replications. Mycelia were harvested from the inoculated peanuts’ seed coats after 10 days cultivation. An amount of 1 g samples (the mixture of peanut seed coat and *A. flavus* mycelia) were grounded to powder after treated by liquid nitrogen, then 600 μL lysis buffer was added, and then the RNA was extracted as per the manufacturer’s instructions (Aidlab, Beijing, China). Genomic DNA was removed by DNase I (Takara, Dalian, China), and RNA quality was evaluated by NanoDrop 2000 spectrophotometer (Thermo Fisher, Waltham, MA, USA) and Agilent 2100 Bioanalyzer (Agilent, Santa Clara, CA, USA).

### 5.5. RNA Sequencing and Transcriptome Processing

The mRNA was sequenced by Novogene (Beijing, China). Briefly, mRNA was purified from total RNA with oligo-dT magnetic beads. The non-strand-specific libraries were constructed by NEB Next Ultra^TM^ RNA Library Prep Kit for Illumina (NEB, USA), and sequenced by the Illumina Hiseq 4000 platform (Illumina Inc., San Diego, CA, USA). Clean reads were harvested by removing the low-quality reads and adaptor, and then mapped to the reference genome (BioProject: PRJNA13284) with HISAT 1.31 [44]. The read counts were used to assess genes’ transcriptions [45]. The differentially expressed genes (DEGs) were evaluated with *p*_adj_ ≤ 0.05 and log_2_ratio ≥ 1 or ≤1. The Gene Ontology (GO) functional analysis and Kyoto Encyclopedia of Genes and Genomes (KEGG) pathway analysis of DEGs were performed with the FungiFun and KAAS, respectively [46,47].

### 5.6. RT-qPCR Analysis

Total RNA was used for reverse transcription, and cDNA synthesis was with a two-step cDNA synthesis kit (TaKaRa, Dalian, China). The Analytic Jena Q-tower system (Analytik-Jena, Jena, Germany) was used for qPCR assays with the 20 μL reaction system, including 5 μL cDNA product, 0.5 μL of each primer, and 10 μL SYBR Green mix (TaKaRa, Dalian, China). All primers are listed in Table S2. The qPCR program was settled as before, which is one cycle of 3 min at 95 °C followed by 40 cycles of 10 s at 95 °C and 40 s at 65 °C, and the melting curve was analyzed from 60 °C to 90 °C with 0.5 °C incremental increases. The internal reference was used with *actin*. The transcriptional expression was based on the CT value, and the differences were calculated with the 2^−ΔΔCT^ method.

### 5.7. Statistical Analysis

Three biological replicates were performed for all experiments. The means with standard deviations represented the results. AFB_1_ yields and conidia productions in different treatments were calculated with one-way analysis of variance (ANOVA) by SPSS 18.0, and statistical differences were evaluated by Tukey’s test with *p* < 0.05. Student’s *t* test was applied in RT-qPCR with * *p* < 0.05 and ** *p* < 0.01.

## Figures and Tables

**Table 1:** Comparisons of AF biosynthesis cluster genes in different *a*_w_ by transcriptome analysis.

Gene_ID (AFLA_)	Gene	Gene Function	Log_2_ (90/95)	Log_2_ (90/99)	Log_2_ (95/99)
139100	*aflYe*	Ser-Thr protein phosphatase family protein	−0.45	−1.21	−0.78
139110	*aflYd*	sugar regulator	−0.86	−0.33	0.50
139120	*aflYc*	glucosidase	−0.42	−0.59	−0.19
139130	*aflYb*	putative hexose transporter	−0.13	−0.59	−0.48
139140	*aflYa*	NADH oxidase	3.94 *	4.05 *	0.09
139150	*aflY*	hypothetical protein	4.96 *	5.20 *	0.24
139160	*aflX*	monooxygenase	4.58 *	5.92 *	1.33
139170	*aflW*	monooxygenase	4.42 *	6.23 *	1.81 *
139180	*aflV*	cytochrome P450 monooxygenase	5.33 *	12.53 *	7.18 *
139190	*aflK*	VERB synthase	4.79 *	11.23 *	6.43 *
139200	*aflQ*	cytochrome P450 monooxigenase	5.14 *	11.79 *	6.65 *
139210	*aflP*	O-methyltransferase A	5.05 *	11.05 *	5.99 *
139220	*aflO*	O-methyltransferase B	5.03 *	12.05 *	10.83 *
139230	*aflI*	cytochrome P450 monooxigenase	6.21 *	13.05 *	6.95 *
139240	*aflLa*	hypothetical protein	5.40 *	14.05 *	8.11 *
139250	*aflL*	P450 monooxygenase	4.73 *	13.77 *	9.03 *
139260	*aflG*	cytochrome P450 monooxygenase	4.22 *	6.17 *	1.94 *
139270	*aflNa*	hypothetical protein	0.83	1.32	0.48
139280	*aflN*	monooxygenase	4.05*	7.46 *	3.39 *
139290	*aflMa*	hypothetical protein	4.30 *	9.85 *	5.53 *
139300	*aflM*	ketoreductase	4.53 *	12.29 *	7.74 *
139310	*aflE*	NOR reductase	4.34 *	7.97 *	3.63 *
139320	*aflJ*	esterase	4.06 *	6.95 *	2.89 *
139330	*aflH*	short chain alcohol dehydrogenase	3.64 *	5.06 *	1.41
139340	*aflS*	pathway regulator	0.54	3.51 *	0.96
139360	*aflR*	transcription activator	0.43	1.82 *	1.37
139370	*aflB*	fatty acid synthase beta subunit	1.22	2.59 *	1.36
139380	*aflA*	fatty acid synthase alpha subunit	1.73	2.06 *	0.31
139390	*aflD*	reductase	3.35 *	3.73 *	0.37
139400	*aflCa*	hypothetical protein	4.19 *	4.46 *	0.26
139410	*aflC*	polyketide synthase	2.85 *	2.73 *	−0.14
139420	*aflT*	transmembrane protein	−0.10	0.22	0.31
139430	*aflU*	P450 monooxygenase	−0.83	0.15	0.96
139440	*aflF*	dehydrogenase	−0.61	−0.16	0.44

Transcriptome analyses were performed in three biological replicates. Data were calculated with read counts. The values 90/95, 90/99, and 95/99 represented the comparisons of *a*_w_ 0.90 vs. 0.95, *a*_w_ 0.90 vs. 0.99, and *a*_w_ 0.95 vs. 0.99, respectively. Significances were marked as * with *p*adj < 0.05 and log_2_ratio ≥ 1 or ≤1.

**Table 2:** Comparisons of conidia-development-related genes in different *a*_w_ by transcriptome analysis.

Gene_ID (AFLA_)	Gene Annotation	Log2 (90/95)	Log_2_ (90/99)	Log_2_ (95/99)
044790	conidiation-specific family protein	0.42	3.54 *	3.11 *
044800	conidiation protein Con6, putative	3.18 *	8.32 *	5.13 *
083110	conidiation-specific protein (Con10), putative	2.78 *	6.32 *	3.54 *
098380	conidial hydrophobin RodA/RolA	6.49 *	8.68 *	2.18 *
014260	conidial hydrophobin RodB/HypB	3.21 *	3.10 *	−0.13
006180	conidial pigment biosynthesis oxidase Arb2/brown2	5.76 *	6.39 *	0.61
016140	conidial pigment biosynthesis scytalone dehydratase Arp1	−1.57	−1.47	−0.08
079710	conidiophore development protein HymA	−0.01	0.88	0.87
082850	C2H2 type conidiation transcription factor BrlA	3.62 *	5.90 *	2.27 *
029620	transcription factor AbaA	4.19 *	2.52 *	−1.69
134030	developmental regulator FlbA	−0.11	−1.10	−1.01
137320	C2H2 conidiation transcription factor FlbC	−1.10	1.12	1.21
080170	MYB family conidiophore development protein FlbD	−0.60	−0.87	0.28
026900	developmental regulator VosA	2.45 *	1.42 *	−1.05
046990	APSES transcription factor StuA	0.24	1.07	0.81
052030	developmental regulatory protein WetA	2.10 *	2.60 *	0.48
101920	extracellular developmental signal biosynthesis protein FluG	0.06	0.40	0.32

Transcriptome analyses were performed in three biological replicates. Data were calculated with read counts. The values of 90/95, 90/99, and 95/99 represented the comparisons of *a*_w_ 0.90 vs. 0.95, *a*_w_ 0.90 vs. 0.99, and *a*_w_ 0.95 vs. 0.99, respectively. Significances were marked as * with *p*adj < 0.05 and log_2_ratio ≥ 1 or ≤1.

**Table 3:** Comparisons of different TFs in different *a*_w_ by transcriptome analysis.

Gene ID (AFLA_)	Gene Description	log2 (90/95)	log2 (90/99)	log2 (95/99)
013240	C6 transcription factor, putative	−2.41	−2.10 *	0.30
015790	C6 transcription factor (Leu3), putative	0.19	1.96 *	1.74
021930	C6 transcription factor RosA	0.53	1.74 *	1.19
023040	C6 transcription factor, putative	−3.02	−4.27 *	−1.25
025720	C6 transcription factor NosA	2.46	2.46 *	2.21 *
029620	transcription factor AbaA	4.19 *	2.52 *	−1.69
030580	C2H2 transcription factor PacC, putative	−0.50	−2.02 *	−1.53
031790	bZIP transcription factor (MeaB), putative	−0.56	−1.80 *	−1.26
033480	C6 transcription factor, putative	1.02	1.85 *	0.81
035590	C6 transcription factor, putative	−0.16	2.75 *	2.25
040300	C6 transcription factor, putative	2.36 *	2.75	0.37
051900	zinc knuckle transcription factor (CnjB), putative	0.48	2.73 *	2.23
056780	C6 transcription factor, putative	−0.84	−2.27 *	−1.44
059510	fungal specific transcription factor, putative	−0.95	−1.76 *	−0.84
070970	C6 transcription factor, putative	0.60	1.61 *	1.00
074200	C6 transcription factor, putative	−0.76	−1.90 *	−1.16
076320	C6 transcription factor, putative	1.24	2.61 *	1.35
078500	bZIP transcription factor, putative	0.92	2.65 *	1.72
082850	C2H2 type conidiation transcription factor BrlA	3.62 *	5.90 *	2.27 *
083460	C6 transcription factor RosA-like, putative	−1.64	−1.91 *	−0.28
083560	C6 transcription factor, putative	0.72	2.01 *	1.28
084720	C6 transcription factor, putative	0.68	2.56 *	1.87
085880	BTB domain transcription factor, putative	1.14	1.42 *	0.27
087810	bZIP transcription factor, putative	0.51	2.69 *	2.17
094010	bZIP transcription factor (*Atf21*), putative	1.06	3.69 *	2.60 *
095090	C6 transcription factor, putative	1.87	5.79 *	3.90
109220	C6 transcription factor, putative	0.77	1.95 *	1.16
Novel00457	fungal specific transcription factor [*Aspergillus oryzae* RIB40]	1.72 *	2.25 *	−0.52
Novel00611	transcription factor [*Aspergillus oryzae* RIB40]	−1.08	−3.22 *	−2.16

Transcriptome analyses were performed in three biological replicates. Data were calculated with read counts. The values of 90/95, 90/99, and 95/99 represented the comparisons of *a*_w_ 0.90 vs. 0.95, *a*_w_ 0.90 vs. 0.99, and *a*_w_ 0.95 vs. 0.99, respectively. Significances were marked as * with *p*adj < 0.05 and log_2_ratio ≥ 1 or ≤1.

## Data Availability

All data are provided in the manuscript.

## Supplementary Materials

The following are available online at https://www.mdpi.com/article/10.3390/toxins13060431/s1, Figure S1: Transcriptional expression analyses of diverse genes by RT-qPCR, Table S1: Comparisons of several global regulators in different *a_w_* by transcriptome analysis, Table S2: Primers used for qPCR analysis.
