# Supplementary material for: The Regulatory Mechanism of Water Activities on Aflatoxins Biosynthesis and Conidia Development, and Transcription Factor AtfB Is Involved in This Regulation
Source: Toxins (Basel). 2021 Jun 21;13(6):431. doi: 10.3390/toxins13060431 (PMC8235239; doi:10.3390/toxins13060431)
Supplement: Supplementary file 1 [file toxins-13-00431-s001.zip › toxins-1174304-supplementary/toxins-1174304 supplementary for conversion .pdf]

Supplementary Materials: The Regulatory Mechanism of Water Activities on Aflatoxins Biosynthesis and Conidia Development, and Transcription Factor AtfB is Involved in This Regulation

Longxue Ma, Xu Li, Xiaoyun Ma, Qiang Yu, Xiaohua Yu, Yang Liu, Chengrong Nie, Yinglong Zhang and Fuguo Xing

Table S1. Comparisons of several global regulators in different a_w by transcriptome analysis.

Gene ID (AFLA_)	Gene	Gene Description	log_2 (90/95)	log_2 (90/99)	log_2 (95/99)
066460	*veA*	developmental regulator AflYf / VeA	0.51	−0.95	−1.48
033290	*laeA*	regulator of secondary metabolism LaeA	−0.53	−0.12	0.39
084190	*velB*	conserved hypothetical protein	0.23	−0.47	−0.72
083380	*pbsB*	MAP kinase kinase (Pbs2) putative	0.42	0.16	−0.28
062500	*maf1*	mitogen-activated protein kinase MAF1	0.22	−0.19	−0.42
103480	*ste7*	MAP kinase kinase Ste7	−0.08	0.02	0.08
035530	*ste20*	serine/threonine kinase Ste20	−0.38	0.28	0.64
048880	*ste11*	MAP kinase kinase kinase Ste11	0.24	0.33	0.07
052570	*mpkA*	MAP kinase MpkA	0.14	0.58	0.42
051240	*map2k*	MAP kinase kinase (Mkk2) putative	−0.19	0.49	0.66
034170	*fus3*	MAP kinase FUS3/KSS1	−0.23	0.14	0.36
031560	*bck1*	MAP kinase kinase kinase (Bck1), putative	0.80	0.47	−0.34
068590	*sskB*	MAP kinase kinase kinase SskB putative	0.03	0.22	0.17
061090	*sakA1*	MAP kinase SakA	−1.38	0.87	2.23
099500	*sakA2*	MAP kinase SakA	−0.94	−1.51 *	−0.59
031340	*atfA*	bZIP transcription factor (AtfA), putative	−0.11	−0.35	−0.25
094010	*atfB*	bZIP transcription factor (Atf21) putative	1.06	3.69 *	2.60 *
129340	*ap-1*	conserved hypothetical protein	−0.27	−0.50	−0.24
110650	*msnA*	C2H2 transcription factor (Seb1) putative	−0.20	0.21	0.38
091490	*mtfA*	C2H2 finger domain protein putative	−0.72	−1.14	0.43
034540	*srrA*	stress response transcription factor SrrA/Skn7, putative	0.50	0.18	−0.34
062210	*sskA*	response regulator putative	0.15	0.44	0.28
026790	*ppoA*	fatty acid oxygenase PpoA, putative	−0.30	−2.59	2.29
120760	*ppoB*	fatty acid oxygenase, putative	6.59 *	12.54 *	5.94 *
030430	*ppoC*	conserved hypothetical protein	0.21	−2.19	−2.41
101920	*fluG*	extracellular developmental signal biosynthesis protein FluG	0.06	0.40	0.32
002850	*AfPXG*	calcium binding protein Caleosin, putative	−0.41	2.24 *	2.63*
025100	*gpdA*	glyceraldehyde 3-phosphate dehydrogenase GpdA	−0.43	0.29	0.71
046760	*gfdB*	glycerol 3-phosphate dehydrogenase (GfdB) putative	−0.75	0.81	1.54
060740	*gprA*	mating-type alpha-pheromone receptor PreB	0.94	0.84	−0.12
061620	*gprB*	a-pheromone receptor PreA	−0.51	0.66	1.15
074150	*gprC*	conserved hypothetical protein	−0.75	−0.94	−0.21
135680	*gprD*	G protein-coupled receptor GprD	−0.96	0.10	1.05
006880	*gprF*	PQ loop repeat protein	0.05	−0.13	−0.20
067770	*gprG*	PQ loop repeat protein	1.39	1.28	−0.13
006920	*gprH*	cAMP receptor-like protein, putative	2.03 *	0.36	−1.69
127870	*gprJ*	vacuolar membrane PQ loop repeat protein	0.55	−0.83	−1.40
009790	*gprK*	conserved hypothetical protein	0.98	0.22	−0.78

075000	*gprM*	conserved hypothetical protein	−1.06	−1.35	−0.32
032130	*gprO*	hemolysin-III channel protein Izh2 putative	−0.23	−0.08	0.14
088190	*gprP*	IZH family channel protein (Izh3) putative	−0.42	0.03	0.43
023070	*gprR*	integral membrane protein	0.11	1.51 *	−1.39
006320	*gprS*	PQ loop repeat protein	−0.02	0.84	0.84
018340	*fadA*	G-protein complex alpha subunit GpaA/FadA	0.02	−0.18	−0.21
093240	*sfaD*	G-protein complex beta subunit SfaD	0.03	−0.19	−0.24
032870	*pkaR*	cAMP-dependent protein kinase regulatory subunit PkaR	0.13	1.54 *	1.40
135040	*pkaC*	cAMP-dependent protein kinase catalytic subunit PkaC1	0.07	1.76 *	1.67 *
018930	*capA*	adenylyl cyclase-associated protein (cap)	−0.16	−0.24	−0.09
071410	*somA*	cAMP-dependent protein kinase pathway protein (Som1)	0.37	0.25	−0.14
112560	*sok1*	cAMP-mediated signaling protein Sok1 putative	0.80	−0.10	−0.92
134680	*creA*	C2H2 transcription factor (CreA) putative	−0.24	0.45	0.68
049870	*areA*	GATA transcriptional activator AreA	0.67	1.74 *	1.06
030580	*pacC*	C2H2 transcription factor PacC putative	−0.50	−2.02 *	−1.53
012010	*farB*	C6 transcription factor (Ctf1B) putative	−1.27	−1.30	0.06

Transcriptome analyses were performed three biological replicates. Data was calculated with read counts. 90/95, 90/99 and 95/99 represented the comparisons of a_w 0.90 vs 0.95, a_w 0.90 vs 0.99 and a_w 0.95 vs 0.99, respectively. Significances were marked as * with *p*adj < 0.05 and log_2ratio ≥ 1 or ≤1.

Table S2. Primers used for qPCR analyses.

**Gene Name**	Gene ID (AFLA_xxx)	Primers		Fragment Length (bp)
aflA	139380	F: aattgctcaacttcctacc	R: ttgggttggcctcgccaaag	143
aflC	139410	F: ttcgaaccgctctagtgtgcc	R: agcgactgcagcttaccgcc	140
aflK	139190	F: tgtcccaggatggaactgcttc	R: ttgataccagtcaaatagagg	152
aflO	139220	F: agcagtggacccctacaag	R: tccgaagaatgcgaccaagg	151
aflV	139180	F: taaggtccctggtccctgg	R: atcggctatgtcaacctcc	151
aflR	139360	F: aggagaaaccggcctgtgctcg	R: aagtccctgttccgacctgg	158
aflS	139340	F: tgaccatctccgacccgttc	R: acgccagcacctggaactcc	154
veA	066460	F: tgcggtgcaggtgcaaagtcc	R: tttccaaagtggcgtacagg	160
atfA	031340	F: ttcgcttgtctaatataacc	R: agtcttcagtttccaaagcg	148
atfB	094010	F: tctcacagcctgacctgatgg	R: aaggcccctctccatgttggg	143
ppoB	120760	F: tgtggagagatctccgcgcc	R: aagctggatgatgcgttcc	143
AfPXG	002850	F: acggattcccgcgactcacatc	R: ataggacgtgctgctggagag	149
con6	044800	F: tggctacaaggccaccctc	R: tcggatccttgttctgctcacc	140
con10	083110	F: tcagcaaccgtccccacgagg	R: accgggtttgaagcttccgc	152
rodA	098380	F: tggcgctggccagcaagtcg	R: agcaaggagaccctcctc	166
rodB	014260	F: tgtcgctgctctcttcgcc	R: agcagacggggtggttgcc	164
brlA	082850	F: agcttctcgcccatggattcc	R: tagacggaaagcactccgc	153
abaA	029620	F: accagcagaccgttcgaatgg	R: agtaagattgcaccggcc	140
wetA	052030	F: agcagtcacccatcatctcc	R: atgaacggtgtcttgaaatg	137
flbA	134030	F: atctctcggccattggagg	R: tccgataatggtggagtcag	146
stuA	046990	F: tcgcagccgccaatctcgg	R: accagccgctaagtccgcag	153
actin	055230	F: ctttgctggcgatgatgctc	R: tggagtgaaaggacaccacg	138

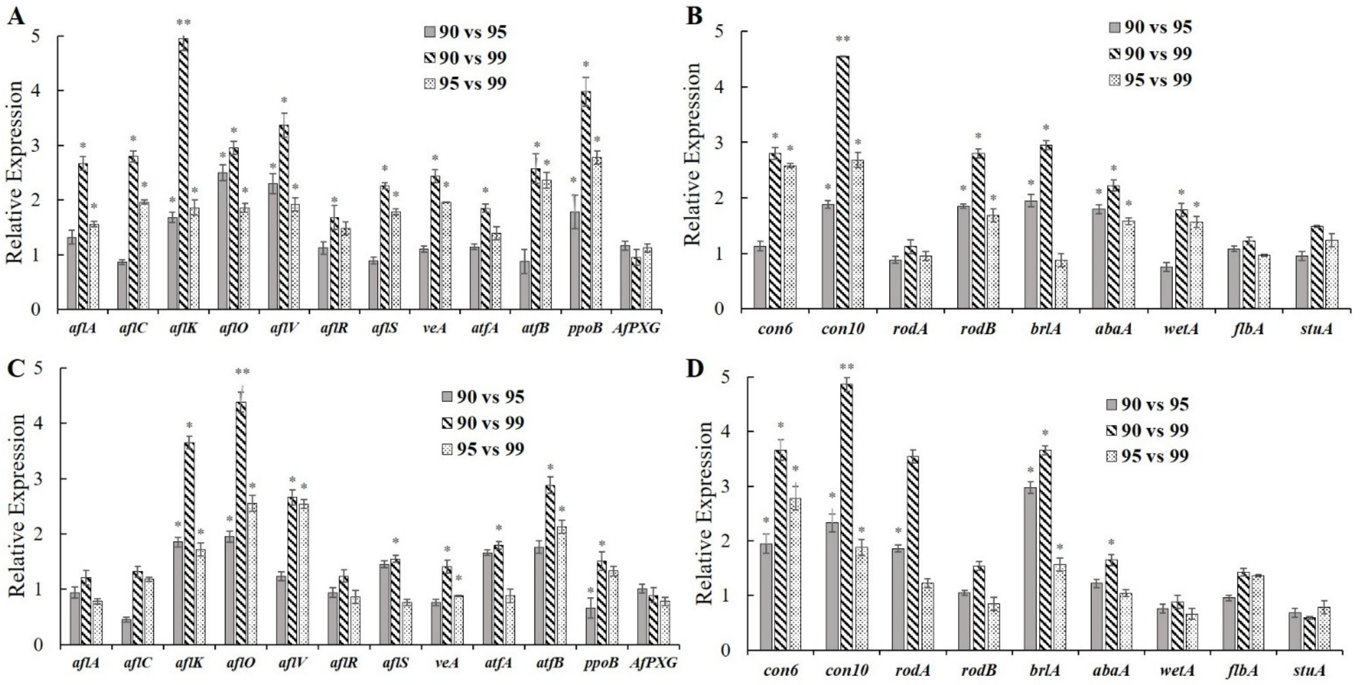

Figure S1. Transcriptional expressions analyses of diverse genes by RT-qPCR. The RT-qPCR analysis of (**A**) AF biosynthesis related genes and (**B**) conidia developmental genes in different a_w conditions in *A. flavus* CA14. The RT-qPCR analysis of (**C**) AF biosynthesis related genes and (**D**) conidia developmental genes in different a_w conditions in *A. flavus* ACCC 32656. The different a_w comparisons were showed as diverse bars. Three independent biologic replicates were performed in each condition, and data were presented as means ± SD. *t* tests were applied for significance analyses with * $p < 0.05$ and ** $p < 0.01$.
